# Poisoning by Tinned Fruit

**Published:** 1890-08

**Authors:** 


### POISONING BY TINNED FRUIT.

Four cases of tin poisoning caused by tinned cherries are reported in London, Eng. It is stated that on January 17, a tin of meat and a tin of cherries were opened, and the contents partaken of at the same time by four men, each of whom ate both meat and cherries and drank some of the cherry juice. One and a half to two hours later they were all attacked with symptoms of irritant poisoning. Three of the cases were admitted in the North-West London Hospital, and were diagnosed as cases of either tin or ptomaine poisoning, and were treated accordingly. The subsequent analyses of the contents of the two tins indicated that the toxic symptoms were due to tin. The meat was found on analysis to be free from tin and from ptomaines. The tin of cherries was about half-full of cherries and juice. The juice was strongly acid, and the analysis of it showed that the acidity was due to malic acid with a small quantity of acid tartrate of potash. The juice contained tin in solution, a quantitative estimation of which showed that every fluid ounce of the juice contained the equivalent of 1.9 grains of the higher oxide of tin, which would be equal to 3.2 grains of the malate of tin in each fluid ounce of the juice, or to 60.4 grains in a pint of the juice.

